# Immediate switching from a chrono to an extended-release formulation of divalproex sodium: A two-part study in patients with controlled epilepsy

**DOI:** 10.4103/0972-2327.41886

**Published:** 2008

**Authors:** Rakesh Shukla, Sanjay Prakash, Asad Abbas

**Affiliations:** Department of Neurology, Chhatrapati Shahuji Maharaj Medical University (Erstwhile King George's Medical University), Lucknow - 226 003, India; 1 Department of Neurology, Government Medical College, Baroda, India

Sir,

Compliance to the prescribed therapy is important in the treatment of epilepsy and is inversely proportional to the number of drug doses per day.[1,2] Most physicians have an apprehension that extended-release (ER) formulations may not maintain the blood levels of anti-epileptic drugs (AEDs) throughout the day. Previous studies have suggested that the total daily dose of sodium valproate is required to be increased by 8–20% while switching from the chrono to the ER formulation.[3,4]

We carried out an open-label prospective study to examine the efficacy of the ER sodium valproate in the management of epilepsy. The study was conducted in two parts. We included patients with epilepsy who were administered a stable mono therapy for at least six months, those administered a chrono preparation of sodium valproate twice a day and those with no seizures in the previous three months. Subjects receiving poly therapy, those with a history of status epilepticus and pregnant or lactating females were excluded.

In part I of the study, the daily dose of the ER divalproate was 25% more than that of chrono preparation of sodium valproate, while in part II of the study, the daily dose of divalproex sodium ER was similar to the total daily dose of chrono preparation of sodium valproate. The patients under study were prescribed a single dose of the commercially available ER divalproate in the morning and were advised to record any adverse effects or seizure recurrence in a diary. The patients were evaluated on day 1 (first visit), day 7 (second visit) and day 90 (third visit). A predose (trough) blood level of sodium valproate in the patients was estimated on all three visits by chemiluminescence technique.[5,6]

In part I study, 24 patients were enrolled of which 18 completed the study. In part II study, 20 patients were enrolled of which 19 completed the 3-month follow-up visit. The demographic characteristics of the study population are given in Table 1. In part I study, one patient had a generalized seizure after being administered the ER preparation, which was probably related to sleep deprivation. The remaining 17 patients were seizure free during the 3-month follow-up. In part II study, none of the 19 patients had any seizure during the 3-month follow-up.

**Table 1:** Demographic characteristics

Clinical features	Study Part I n = 18	Study Part II n = 19
Age (years)
Mean ± SD	23 ± 11	24 ± 12
Range	12–34	12–62
Sex
M:F	12:6	16:3
Type of seizure
Partial	4	–
Generalized	14	19
Seizure-free duration (months)
Mean ± SD	28 ± 17.5	23 ± 18.6
Range	3–60	3–72
Head CT scan
Normal	16	17
Abnormal*	2	2
EEG
Normal	14	13
Abnormal	4	6

* A calcified dot lesion was seen.

The serum valproate levels are depicted in Table 2. In part I study, serum valproate levels were significantly higher on days 7 and 90 under ER divalproate treatment than the baseline levels on day 1 under treatment with the chrono preparation. The higher blood levels could be attributed to the 25% increase in the total daily dose of the drug during switching from the chrono to the ER preparation. It has been reported that ER divalproex sodium has a lower bioavailability than the conventional delayed-release formulation; however, studies recommending a higher divalproex sodium ER daily dose were performed on normal healthy volunteers.[3,4,7] Whether these apply to clinical settings such as epilepsy require further studies.

**Table 2:** Serum level and dose of valproate used

Visit/Day@	Serum valproate mean ± SD	Levels (ug/ml) range	Daily valproate mean ± SD	Dose (mg) range
Part 1
Day 1	54.7 ± 26.1	26.7–84.0	694 ± 277	400–1000
Day 7	83.1 ± 27.8*	34.3–147.0	826 ± 244	500–1125
Day 90	74.3 ± 20.5**	48.7–119.0	826 ± 244	500–1125
Part II
Day 1	68.8 ± 25.7	22.4–111.7	858 ± 278	250–1300
Day 7	66.1 ± 31.1	23.9–128.7	862 ± 276	250–1250
Day 90	65.9 ± 25.9	26.2–104.3	855 ± 268	500–1250

@ On visit 1, patients were taking chrono preparation, while on visits 2 and 3, they were taking ER preparation. Significance level

* 1%

** 5% by Wilcoxon signed rank test.

In part II study, wherein the total daily dose of the ER divalproate and chrono preparation were the same, no significant difference was observed in serum valproate levels on days 7 and 90 as compared to that on day 1. The baseline serum valproate level on day 1 while the patient was administered chrono preparation was measured approximately 12 h after the last dose; on the other hand, those on days 7 and 90 while the patients were taking ER preparation were done approximately 24 h after the last dose. Studies in persons with epilepsy[5] and psychiatric disorders[6] also have shown that ER single dose divalproate (without additional increment in daily dose) can maintain the same or higher blood level of valproate as achieved with delayed ER formulations. Unlike a previous study conducted on a small sample size (5 subjects) for a shorter duration (60 days), our study was conducted on a larger number of subjects (20 patients) for a longer duration (90 days). Fallacies in the timing of blood sampling (trough versus peak) could be attributed to the relatively lower blood levels of valproate reported in some of the previous studies where the blood levels achieved with the ER (single dose) have been compared with delayed-release (DR; twice daily dose) formulations.[7]

The patients had significantly less headache while on ER formulation than that while on DR formulation. It is possible that the improvement in headache may be associated with more stable blood levels of the drug achieved with the ER formulation. Tremor of hands, gastric upset and aggression were less during the intake of ER preparation than that during the intake of DR preparation in both studies; however, there was no significant difference between the two formulations. There was no significant change in body weight (49.9 kg on chrono preparation and 49.1 kg on ER preparation in part I study; 50.1 kg on chrono preparation and 50.4 kg on ER preparation in part II study). Our results indicate that switching over from the chrono to ER formulation without dose escalation is safe and effective.
